# Design and TAG-Assisted Synthesis of the C-Terminal Amidated Antimicrobial Peptide NCBP-1 Derived from a Plant-Derived Noncanonical NCBP and Its Biological Activity

**DOI:** 10.2174/0109298665465381260330113137

**Published:** 2026-04-24

**Authors:** Ziying Geng, Linyan Zhang, Haidi Li, Taigang Liang

**Affiliations:** 1 Medicinal Basic Research Innovation Center of Chronic Kidney Disease, Ministry of Education, School of Pharmacy, Shanxi Medical University, Taiyuan, Shanxi, 030001, China;; 2 Shanxi Provincial Key Laboratory of Drug Synthesis and Novel Pharmaceutical Preparation Technology, School of Pharmacy, Shanxi Medical University, Taiyuan, Shanxi, 030001, P.R. China

**Keywords:** NCBP, Tag-assisted peptide synthesis (TAPS), antibacterial activity, anti-inflammatory activity, molecular docking, antimicrobial peptides (AMPs)

## Abstract

**Introduction:**

The rise in global bacterial resistance necessitates the discovery of novel antibiotics. Plant-derived Antimicrobial Peptides (AMPs) offer structural diversity and biocompatibility. This study aims to investigate the green synthesis and biological activities of derivatives of NCBP, a linear non-classical AMP identified from plants.

**Methods:**

Five NCBP derivatives (NCBP-1 to NCBP-5) were generated using a green tag-assisted peptide synthesis (TAPS) strategy, combined with site-directed mutagenesis and terminal modification. The peptides were characterized by MS and HPLC and subsequently evaluated for antibacterial activity against ten bacterial strains, salt tolerance, and cytotoxicity in RAW 264.7 murine macrophages. Molecular docking was performed to assess binding interactions.

**Results:**

NCBP-1 was identified as the lead derivative, demonstrating potent antibacterial activity (MIC 8 μg·mL^−1^) and low cytotoxicity. It also exhibited moderate anti-inflammatory activity in LPS-stimulated RAW 264.7 macrophages. Its antibacterial mechanism was further supported by favorable molecular docking interactions with *E. coli* outer membrane LPS (PDB ID: 4RHB).

**Discussion:**

The combined approach successfully identified NCBP-1 as a potent antibacterial candidate. Its activity against Gram-negative bacteria is likely related to LPS binding, as suggested by the docking results. Further studies would be needed to fully elucidate its mechanism of action.

**Conclusion:**

NCBP-1 represents a promising lead for the development of novel antibacterial agents, particularly for treating Gram-negative bacterial infections.

## 1 INTRODUCTION

In recent years, the global spread of bacterial resistance has emerged as a critical threat to public health [1-5]. Notably, the increasing incidence of infections caused by Multidrug-Resistant (MDR) Gram-negative bacteria (*e.g., Escherichia coli* and *Shigella spp*.) and clinically relevant resistant strains, such as methicillin-resistant *Staphylococcus aureus* (MRSA), has drastically reduced the therapeutic efficacy of conventional antibiotics, highlighting an urgent demand for the development of novel antimicrobial agents [6-8]. Against this backdrop, antimicrobial peptides (AMPs) have emerged as promising alternatives to traditional antibiotics, owing to their distinct mechanisms of action (*e.g.,* disruption of bacterial membrane integrity) and reduced tendency to elicit resistance [9-11].

As a vital subset of natural bioactive peptides, plant-derived AMPs exhibit inherent advantages including structural diversity and excellent biocompatibility, rendering them a major focus of contemporary antimicrobial research [12-14]. NCBP is a linear non-classical antimicrobial peptide composed of 11 amino acid residues (Sequence: KPWLRVALCPG), which was originally identified *via* a plant peptide genomics approach [15]. Preliminary investigations have demonstrated that NCBP exhibits moderate antimicrobial activity; however, its efficacy, particularly against Gram-negative bacteria, and clinical safety profile still require further optimization.

The bioactivity of AMPs is tightly linked to their structural characteristics, with key contributing factors including net positive charge, hydrophobicity, and secondary structure [10, 16-20]. To improve the antibacterial efficacy of NCBP, well-established strategies in AMP research, such as site-directed mutagenesis and terminal modification, have been adopted in this study [21-23]. For example, the introduction of positively charged Lysine (Lys) residues can enhance electrostatic interactions with bacterial membranes [24-26], specifically the lipopolysaccharide (LPS) layer of Gram-negative bacteria, thus facilitating membrane penetration [27-29]. C-terminal amidation mitigates interference from negative charges, thereby enhancing peptide stability and improving antimicrobial efficacy [30-33]. Furthermore, substituting residues within the hydrophobic core (*e.g.,* replacing Alanine (Ala) with Tryptophan (Trp) can enhance membrane binding *via* increased hydrophobicity, which in turn further potentiates antibacterial activity [34-38].

Guided by this rationale, the present study aims to rationally design NCBP derivatives to systematically optimize their antimicrobial efficacy and biosafety profiles. Specifically, five NCBP derivatives (NCBP-1 ~ NCBP-5) were constructed *via* three types of structural modifications: (1) introduction of Lys residues at the N-terminus or C-terminus to increase net positive charge; (2) C-terminal amidation; and (3) site-directed mutations within the hydrophobic core. Bioinformatics tools, including CAMPR4, DBAASP, and HeliQuest, were used to predict three key aspects of the derivatives: antimicrobial potential, sequence novelty, and critical physicochemical properties [39-44]. These predictions were used to guide the rational design of the peptides. The target peptides (NCBP-1 ~ NCBP-5) were synthesized *via* green TAG-Assisted peptide synthesis (TAPS) strategy [45-51], purified using reversed-phase high-performance liquid chromatography (RP-HPLC), and characterized by mass spectrometry (MS) to verify their purity and structural identity. The Minimal Inhibitory Concentrations (MICs) of the peptides (NCBP-1 ~ NCBP-5) against ten strains of gram-positive and gram-negative bacteria, including MRSA, were determined using the microbroth dilution method. Cytotoxicity of the peptides toward RAW 264.7 murine macrophage cells was evaluated using the Cell Counting Kit-8 (CCK-8) assay. Additionally, the anti-inflammatory activity in lipopolysaccharide (LPS)-stimulated RAW 264.7 macrophages was evaluated. Furthermore, the antibacterial activity of NCBP-1 was further validated *via* molecular docking with the outer membrane LPS (PDB ID: 4RHB) of *E coli*.

By integrating rational structural modification and systematic bioactivity screening, the present study seeks to identify novel antimicrobial peptide candidates with enhanced efficacy and reduced cytotoxicity. These candidates are expected to serve as potential therapeutic alternatives for combating Gram-negative bacterial infections and to provide a theoretical basis for the molecular engineering of plant-derived AMPs.

## 2 MATERIALS AND METHODS

### 2.1 Design and Prediction of Peptides

Bioinformatics-based prediction and analysis tools were used to assess the potential of various amino acid sequences to function as AMPs. We selected the five peptides with the highest probability of having low MIC scores in CAMPR4, DBAASP, and HydrAMP databases as potential active AMPs. The sequences of the five peptides exhibited no significant similarities to any peptide in the APD3, CAMPR3, and DRAMP databases, confirming their novelty. Finally, the physico-chemical characteristics of the promising AMPs were identified by online software: mean hydrophobicity, mean hydrophobic moment, net positive charge, and helical wheel projections.

### 2.2 Synthesis and Characterization of NCBP Peptides

The synthesis of peptides NCBP-1~NCBP-5 was assisted by the phosphorus-containing C-terminal amidation tag Rink Amide-TAG, which was previously established by our research group [52-58], as well as the research on the synthesis and structure-activity relationships (SAR) of antimicrobial cyclopeptides [59]. TAG-assisted peptide synthesis (TAPS) strategy integrates the merits of traditional liquid-phase peptide synthesis (LPPS) and solid-phase peptide synthesis (SPPS), facilitating homogeneous reactions while obviating the requirement for chromatography. The peptides were purified *via* reversed-phase high-performance liquid chromatography (RP-HPLC). All purifications were performed on a YMC-Trart C18 (4.6 × 250 mm, S-5 μm, 12 nm). A nonlinear water/acetonitrile gradient containing 0.1% trifluoroacetic acid was employed for 30 min, with a flow rate of 1.0 mL·min^−1^. In this study, the purity of all synthesized peptides exceeded 95%. The identity of all peptides was confirmed by liquid chromatography-mass spectrometry (LC-MS; Waters ZQ2000). All synthesized peptides possess a C-terminal amide group.

### 2.3 Antimicrobial Activity

The peptides were dissolved in sterile dimethyl sulfoxide (DMSO) to a final concentration of 12.8 mg/mL. The test bacterial strains were first cultured on Mueller-Hinton agar (MHA), selected based on colony morphology, and then inoculated into Mueller-Hinton broth (MHB) and incubated at 37^o^C for 16 h. The resulting bacterial cultures were adjusted to a 0.5 McFarland standard (MCF) with MHB and then diluted 1:100 for use in the experiment. Bacterial suspensions were co-incubated with an equal volume of peptide solutions at different concentrations (2-64 μg/mL). After incubation at 37^o^C for 18 h, the Optical Density (OD) of each sample was measured at 600 nm using a microplate reader. Polymyxin B (for Gram-negative bacteria) and vancomycin (for Gram-positive bacteria) were used as positive controls. The MICs of the peptides against ten bacterial strains (including *Staphylococcus* aureus ATCC 43300, *Escherichia* coli ATCC 25922, and *Shigella* flexneri ATCC 12022) were determined using the aforementioned method. All data were acquired from two independent experiments, each conducted in triplicate. The MIC was defined as the lowest concentration of the peptide that completely inhibited bacterial growth.

### 2.4 Salt Tolerance

Determination of Minimum Inhibitory Concentration (MIC) under inorganic salt conditions. Mueller-Hinton Broth (MHB) supplemented with 4.5 mM KCl, 1 mM MgCl_2_, 150 mM NaCl, and 1 mM CaCl_2_ was prepared. Three to five morphologically consistent single colonies were picked from an MHA (Mueller-Hinton Agar) plate inoculated with *Escherichia coli* ATCC 25922 and inoculated into the aforementioned inorganic salt-supplemented MHB. The culture was subjected to shaken incubation at 37°C and 150 rpm for 18 h. The bacterial suspension was diluted to 1×10^6^ CFU/mL using the same inorganic salt-containing MHB. Meanwhile, the antimicrobial peptides NCBP-1 to NCBP-5 were subjected to 2-fold serial dilutions in inorganic salt-supplemented MHB to generate a concentration gradient (128-4 μg/mL). A total of 100 μL of the bacterial suspension and 100 μL of each peptide dilution were added to a 96-well plate, followed by incubation at 37°C for 18 h to determine the MIC. Each experiment was performed in triplicate and repeated twice independently.

### 2.5 Cytotoxicity Assay

The peptides were dissolved in PBS containing less than 0.1% DMSO to prepare a stock solution (51.2 mg/mL). Prior to use, the stock solution was diluted 100-fold with culture medium, followed by a two-fold serial dilution to obtain the desired working concentrations, ranging from 16 to 256 μg/mL. The experiment was designed with three groups: a blank group, a cell control group, and an antimicrobial peptide-treated experimental group, with four replicate wells established for each group. The blank group was supplemented with 100 μL of cell-free complete culture medium, whereas the cell control group and the experimental group were each inoculated with RAW264.7 cells. In this experiment, RAW264.7 murine macrophages in the logarithmic growth phase were seeded into sterile 96-well cell culture plates at a density of 1 × 10^4^ cells per well, with a total volume of 100 μL per well. The peripheral wells of each 96-well plate were filled with 200 μL of sterile PBS to minimize edge effects. Following 24 hours of incubation at 37^o^C in a humidified 5% CO_2_ atmosphere, the spent medium was carefully aspirated and discarded using a pipette. The experimental group was treated with 100 μL of the antimicrobial peptide working solutions at different concentrations, resulting in final concentrations of 16, 32, 64, 128, and 256 μg/mL in each well. In contrast, the blank group and cell control group were each supplemented with 100 μL of fresh culture medium. After an additional 24 hours of incubation under the same conditions (37^o^C, humidified 5% CO_2_ atmosphere), 10 μL of CCK-8 reagent was added to each well, and the plates were further incubated at 37^o^C for 2 hours. The absorbance (optical density, OD) at a wavelength of 450 nm was measured using a microplate reader. All experiments were performed independently in triplicate.

### 2.6 Anti-inflammatory Activity

With the cell viability maintained above 80%, 64 μg/mL was ultimately determined as the final concentration of the antimicrobial peptides for inhibiting nitric oxide (NO) release. RAW264.7 macrophages in the logarithmic growth phase were seeded into sterile 96-well plates at a density of 2×10^5^ cells/well, with 100 μL of cell suspension added to each well. After incubation at 37^o^C under 5% CO_2_ for 24 h, the supernatant was aspirated and discarded. The cells were randomly divided into three groups: blank, model, and administration, with 4 replicate wells per group. The blank group received 100 μL of basic medium, the model group received 100 μL of 1 μg/mL LPS, and the administration group received 100 μL of a mixed solution containing 1 μg/mL LPS and 64 μg/mL of the peptide. The cells were further incubated at 37^o^C in a 5% CO_2_ atmosphere for 24 h. According to the instructions of the NO detection kit, 50 μL of supernatant was collected from each well, followed by sequential addition of 50 μL of Griess Reagent I and 50 μL of Griess Reagent II. The optical density (OD) was measured at 540 nm.

### 2.7 Molecular Docking of Antimicrobial Peptides with Key Proteins

The structures of the AMPs (NCBP-1 to NCBP-5) were drawn in ChemDraw 20.0 and converted to PDB format using Chem3D. Subsequently, energy minimization of the AMP molecules was performed using SYBYL-X 2.0, based on the Tripos force field, to make the molecular models more consistent with the stable, reasonable conformations of real systems. The X-ray crystal structure (PDB ID: 4RHB) of *E. coli* outer membrane LPS (Crystal structure of the lipopolysaccharide assembly complex LptD-LptE from the *Escherichia coli* outer membrane) was retrieved from the Protein Data Bank (PDB) database (https://www.rcsb.org/). Subsequently, PyMOL (v2.2.0) was used to remove water molecules and excess impurities from the protein structure to ensure an accurate protonation state. In AutoDock Vina (v1.1.2), a cubic grid box with dimensions of 80 × 80 × 80 Å was generated around the aforementioned active site, with a spacing of 0.375 Å, encompassing the specified active site within the set box. All docking simulations were conducted employing a semi-flexible docking approach, with an initial population size of 50 individuals, utilizing the Lamarckian Genetic Algorithm (LGA). Other parameters were maintained at default values. Based on the minimum energy scores of all docking results, the optimal conformation with the lowest binding energy was selected for analysis. The docking results were subjected to 3D visualization analysis using Pymol (v2.2.0) and simultaneously to 2D visualization analysis *via* LigPlot+ (v2.2.9).

## 3 RESULTS AND DISCUSSION

### 3.1 Design and Prediction of Peptides

**Figure 1:**
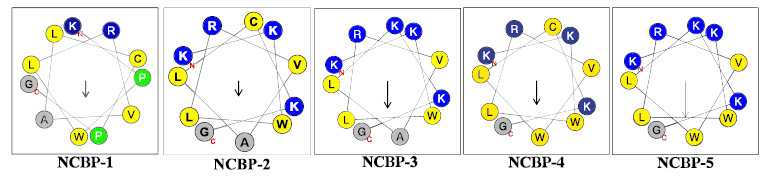
The α-helix of peptides predicted by Heliquest software.

NCBP is a linear, non-classical antimicrobial peptide (AMP) consisting of 11 amino acid residues (Sequence: KPWLRVALCPG), which was successfully identified using the previously reported plant polypeptide genomics approach [15]. To improve the antimicrobial activity of NCBP, we designed a series of its derivatives by introducing additional lysine (Lys) residues at either the N-terminus or C-terminus to increase the net positive charge; all derivatives were further amidated at the C-terminus. NCBP-1 was modified solely by C-terminal amidation, with no other amino acid substitutions or deletions. NCBP-2 was designed by substituting all Proline (Pro) residues at both the N-terminus and C-terminus of NCBP-1 with Lysine (Lys). NCBP-3 was further derived from NCBP-2 by substituting the hydrophobic cysteine (Cys) residue at the C-terminus with Lys. In the helical wheel projection of NCBP-1, the 7th amino acid residue, which is alanine (Ala), is localized in the core region of the hydrophobic face. Thus, NCBP-4 was derived from NCBP-2 by substituting AA7 (Ala) with Trp, while NCBP-5 was derived from NCBP-3 by the same substitution (Tables **1** and **2**, and Figure **1**).

### 3.2 Synthesis and Characterization of NCBP Peptides

**Figure 2:**
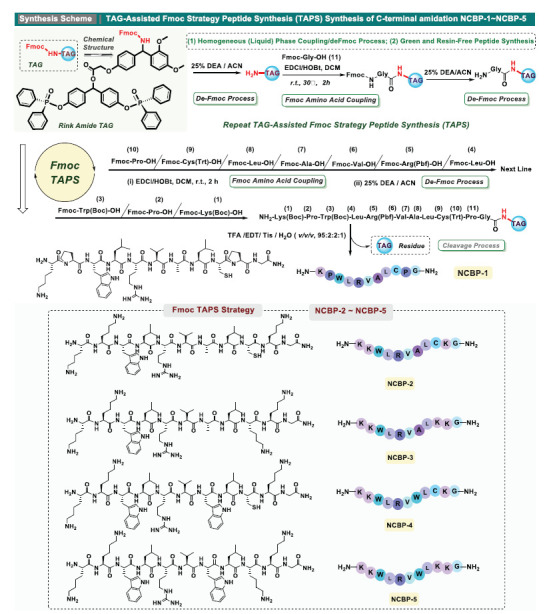
The scheme of the TAG-assisted Fmoc peptide synthesis strategy (TAPS) for the synthesis of the antimicrobial peptide derivatives NCBP-1 to NCBP-5.

**Figure 3:**
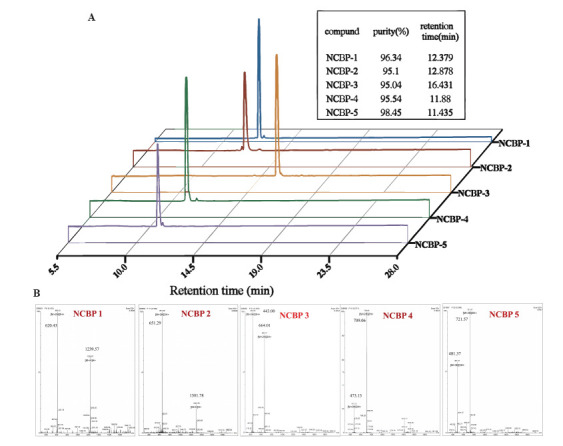
HPLC analysis **(A)** and MS analysis **(B)** of Antimicrobial Peptide NCBP-1 to NCBP-15.

Tag-assisted peptide synthesis (TAPS) strategy enables straightforward purification *via* washing and precipitation, ultimately yielding high-quality peptide products (Figure **2**). The TAPS strategy enables the rapid and efficient acquisition of target peptides NCBP-1 to NCBP-5. Compared with traditional liquid-phase peptide synthesis (LPPS) and solid-phase peptide synthesis (SPPS) strategies, the TAPS strategy reduces the consumption of raw amino acid materials by 2-3 times and cuts down the usage of chemical reagents by 8-10 times. The initial synthesis of NCBP-1 involved the preparation of TAG loaded eleven-peptide chain. Rink Amide-TAG was derived from the esterification of the 4,4’-diphenyl phosphonyloxy diphenylcarbinol tag with the Fmoc Rink Amide group. The soluble small-molecular Rink Amide TAG and the 11^th^ Fmoc amino acid unit, Fmoc-Gly^11^-OH, were amidation-coupled using the coupling reagent system EDCl/HOBt to obtain the TAG-loaded amino acid Fmoc-^11^Gly-NH-Rink-TAG. After the coupling reaction, the product was thoroughly washed with saturated NH_4_Cl and concentrated. Then, an initial precipitation/ purification step was performed using the DCM/PE solvent system. The Fmoc-^11^Gly-NH-Rink-TAG was then deprotected *via* removal of the Fmoc group using the DEA/ACN (v/v, 1:3) system, while the resulting H-11Gly-NH-Rink-TAG was purified by precipitation using a DCM/PE (v/v, 1:10) solvent system. The H-^11^Gly-NH-Rink-TAG obtained above was amidated coupling with the 10^th^ unit Fmoc-^10^Cys(Trt)-OH using the EDCl/HOBt coupling reagent system to synthesize tag-loaded Fmoc-^10^Cys(Trt)-^11^Gly-NH-Rink- TAG. Elongation of the NCBP peptide chain was accomplished through successive Fmoc deprotection and Fmoc-amino acids coupling cycles according to the above procedures. Following the completion of the NCBP intermediate peptide loaded onto Rink amide TAG, the NCBP peptides were cleaved from the Rink amide TAG by means of a cleavage cocktail consisting of Trifluoroacetic Acid (TFA), Ethanedithiol (EDT), Triisopropylsilane (TIS), and deionized water (95:2:2:1, *v/v/v/v*) under constant agitation for 1.5 h. Subsequently, the NCBP peptides were precipitated four times with chilled diethyl ether, followed by lyophilization (Figure **2**). The peptides were purified and identified by RP-HPLC (Figure **3A**) and MS (Figure **3B**).

### 3.3 Antimicrobial Activity

**Figure 4:**
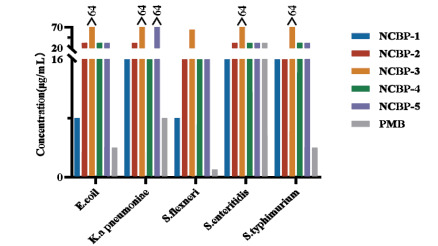
Histogram of the MIC for the synthesized peptides NCBP-1 ~ NCBP-5 against five gram-negative bacteria.

**Figure 5:**
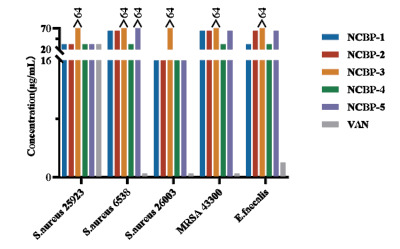
Histogram of the MIC for the synthesized peptides NCBP-1 ~ NCBP-5 against five gram- positive bacteria.

The antimicrobial activities of the five peptides (NCBP1-5) were evaluated and summarized using the minimum inhibitory concentration (MIC) and geometric mean (GM) concentration (Table **3**). We observed that these analogues exhibit stronger antimicrobial activity against Gram-negative bacteria than against Gram-positive bacteria. The MIC values were determined *via* the microbroth dilution method, following the guidelines of the Clinical and Laboratory Standards Institute (CLSI; Wayne, PA, USA; 2013) [60]. Briefly, different concentrations of the NCBP peptides were tested in triplicate to ensure reproducibility, and each concentration was tested in at least two independent experiments. The antimicrobial activity results showed that the MIC values of NCBP-3 against all 10 tested bacterial strains exceeded 128 μg/mL, except for *Shigella* flexneri ATCC 12022, for which the MIC was 64 μg/mL. NCBP-5 exhibited MIC values of 128μg/mL against *Klebsiella pneumoniae* ATCC 13883 and *Staphylococcus aureus* ATCC 6538, whereas it showed an MIC of 16μg/mL against both *S.* flexneri ATCC 12022 and *Staphylococcus* aureus CMCC(B) 26003. NCBP-1, NCBP-2, and NCBP-4 exhibited relatively strong antimicrobial activities against all ten bacterial strains (MICs = 8-64 μg/mL). Among these peptides, NCBP-1 exhibited the most potent inhibitory activity against *Escherichia* coli ATCC 25922 and *S.flexneri* ATCC 12022, with an MIC of 8 μg/mL against both strains. Against the clinical drug-resistant strain methicillin- resistant *Staphylococcus aureus* (MRSA: *Staphylococcus aureus* ATCC 43300), NCBP-4 exhibited the strongest antimicrobial activity, with an MIC of 32 μg/mL (Table **3**, Figure **4**, and Figure **5**).

Notably, from a structure-activity relationship (SAR) perspective, substituting two residues with Lys to enhance the positivity of NCBP peptides fails to achieve the desired effect; additionally, the drastic decrease in the peptide's inhibitory activity after replacing Cys with Lys. The possible reasons were that the outer membrane of Gram-negative bacteria (mainly composed of LPS) is the primary target of AMPs. Cysteine (Cys) has a thiol group (-SH) with strong nucleophilicity and redox activity: it forms hydrogen bonds, hydrophobic interactions, and even covalent bonds with LPS phosphate groups and lipid A fatty acid chains to disrupt the outer membrane, and induces ROS to damage the cell membrane and cause intracellular leakage. In contrast, lysine (Lys) binds LPS *via* weak electrostatic interactions (*via* its positively charged amino group) and lacks redox activity (cannot induce ROS), making it hard to penetrate the outer membrane. This is why replacing Cys with Lys drastically reduces antimicrobial activity. When tryptophan (Trp) is introduced into a peptide sequence, its indole aromatic ring confers both strong hydrophobicity and polarizability. Specifically, the hydrophobic skeleton of the indole ring can form tight hydrophobic interactions with the fatty acid chains of lipid A and the hydrophobic tails of membrane phospholipids, thereby re-endowing the peptide chain with the ability to anchor to the bacterial membrane. Furthermore, the N-H group on the indole ring can form hydrogen bonds with the phosphate groups of LPS and the polar heads of membrane phospholipids, thereby enhancing the peptide's binding specificity to the outer membrane of Gram-negative bacteria. This facilitates the peptide chain's crossing of the outer membrane and its entry into the cell membrane, ultimately disrupting the bacterial cell wall and exerting bactericidal effects.

### 3.4 Salt Tolerance

**Figure 6:**
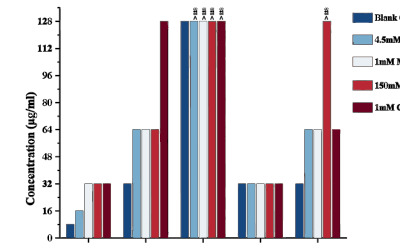
Determination of the minimum inhibitory concentration (MIC) of NCBP-1 to NCBP-5 under inorganic salt conditions.

The results presented the MICs of antimicrobial peptides NCBP-1 to NCBP-5 against *E. coli* ATCC 25922 in the presence of inorganic salts (4.5 mM KCl, 1 mM MgCl_2_, 150 mM NaCl, and 1 mM CaCl_2_) (Figure **6**) NCBP-1 exhibited reduced antibacterial activity against *E. coli* under the influence of these inorganic salts: its activity decreased by 2-fold in the presence of 4.5 mM KCl (MIC increased from 8 μg/mL to 16 μg/mL) and by 4-fold in the presence of 1 mM MgCl_2_, 150 mM NaCl, or 1 mM CaCl_2_ (MIC increased from 8 μg/mL to 32 μg/mL). Similarly, NCBP-2 showed a 2-fold reduction in activity when exposed to 4.5 mM KCl, 1 mM MgCl_2_, or 150 mM NaCl (MIC increased from 32 μg/mL to 64 μg/mL), and a 4-fold reduction in the presence of 1 mM CaCl_2_ (MIC increased from 32 μg/mL to 128 μg/mL). NCBP-3 lost its antibacterial activity in the presence of all four inorganic salts (MIC > 128 μg/mL). Notably, NCBP-4 maintained stable activity under the tested inorganic salt conditions (MIC 32 μg/mL). For NCBP-5, its activity decreased by 2-fold in the presence of 4.5 mM KCl, 1 mM MgCl_2_, or 1 mM CaCl_2_ (MIC increased from 32 μg/mL to 64 μg/mL), while its activity was abrogated in 150 mM NaCl (MIC > 128 μg/mL). Overall, the antibacterial activity of NCBP-1, NCBP-2, NCBP-3, and NCBP-5 against *E. coli* was attenuated in the presence of inorganic salts, whereas NCBP-4 demonstrated excellent salt tolerance.

### 3.5 Cytotoxicity Assay

To evaluate whether the synthesized novel antimicrobial peptides (NCBP-1 ~ NCBP-5) exhibit low cytotoxicity, we used the Cell Counting Kit-8 (CCK-8) assay with the RAW264.7 macrophage cell line as a model to assess their cytotoxicity [61, 62].

**Figure 7:**
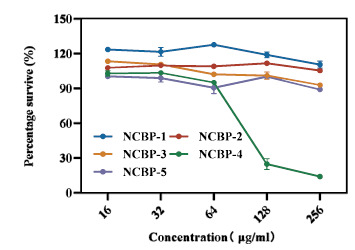
The percentage of RAW 264.7 cell survival was assessed following exposure to antimicrobial peptides NCBP-1 ~ NCBP-5 (concentration range: 16 to 256 μg/mL).

Peptides with no significant cytotoxicity were identified. The results illustrate the effects of these analogues on cell viability at different concentrations (Figure **7**). Derivatives NCBP-1, NCBP-2, NCBP-3, and NCBP-5 exhibited cell viability above 90% even at a concentration of 256 μg/mL, indicating no significant cytotoxicity. In contrast, NCBP-4 exhibited cell viability of less than 30% at 128 μg/mL, suggesting that concentrations above 64 μg/mL inhibit cell growth and metabolism to a certain extent. Overall, the five tested analogues (NCBP-1 to NCBP-5) exhibited relatively low cytotoxicity at concentrations ≤ 64 μg/mL, with cell viability remaining ≥ 90%. This finding supports their potential for further investigation in antimicrobial therapeutic applications.

### 3.6 Anti-inflammatory Activity

Anti-inflammatory activity in LPS-stimulated RAW264.7 macrophages. In this study, an inflammatory model of RAW264.7 cells stimulated by lipopolysaccharide (LPS) was established to evaluate the anti-inflammatory effects of antimicrobial peptides NCBP-1 to NCBP-5.

**Figure 8:**
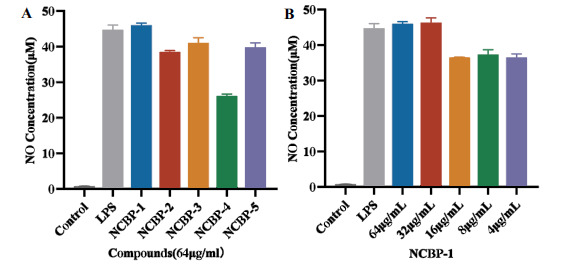
(**A**) The inhibitory effects of antimicrobial peptides NCBP-1~NCBP-5 on LPS-induced NO production. (**B**) The inhibitory effects of NCBP-1 on LPS-induced NO production with various configurations.

After stimulation with LPS at a final concentration of 1 μg/mL, the NO release in the model group exceeded 40 μM. Following the exogenous addition of antimicrobial peptides (64 μg/mL), the levels of inflammatory factors remained relatively stable compared to the model group. This phenomenon may be attributed to the formation of aggregates by high-concentration antimicrobial peptides through interaction with the cell membrane [63-65], which non-specifically perturbs cellular signaling pathways, impairs the normal inflammatory response of cells to LPS, and thus prevents the effective initiation of anti-inflammatory regulation [66]. In the experiment investigating the inhibitory effects of NCBP-1 to NCBP-5 at a series of concentrations on inflammatory factor release (Figure **8A**), low-concentration NCBP-1 may exhibit reduced peptide aggregation, leading to diminished interference with signaling pathways and the restoration of normal cellular responsiveness to LPS (Figure **8B**). Meanwhile, the antimicrobial peptide may exert immunomodulatory effects to inhibit the overexpression of inflammatory factors, thereby reducing NO release [67].

### 3.7 Molecular Docking of Antimicrobial Peptides with Key Proteins

To identify the potential targets of these compounds against *Escherichia coli* (*E. coli*), further molecular docking studies were conducted. Antimicrobial peptides (AMPs) exert their effects by acting on bacterial outer membrane proteins, blocking specific channel proteins, or disrupting the normal function of receptor proteins, thereby affecting bacterial survival [68-70]. However, inhibitors targeting the outer membrane lipopolysaccharide (LPS) structure of *E. coli* are considered effective candidates for the treatment of bacterial infections [71-73]. Molecular docking simulations were performed to investigate the interactions between five AMPs and *E. coli* outer membrane LPS (4RHB), aiming to explore the mechanism of action of AMPs.

**Figure 9:**
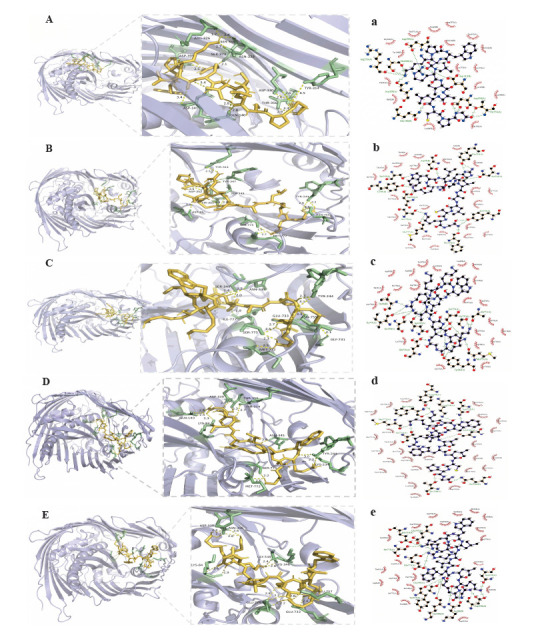
Molecular docking analysis of antimicrobial peptides NCBP-1 ~ NCBP-5. (**A~E**) represent the general overview and local overview for the NCBP1~5 with the 4RHB protein complex. (**a~e**) represent the hydrophobic interactions for the NCBP1~5 with 4RHB protein complex. In the 3D binding poses (left panels), purple indicates protein, yellow indicates antimicrobial peptides, green indicates the amino acid residues where antimicrobial peptides form hydrogen bonds with 4RHB, and yellow dashed lines represent hydrogen bonds. In the 2D interaction diagrams (right panels), green dashed lines represent hydrogen bonds, and radial arcs represent the amino acid residues involved in hydrophobic interactions.

We individually depicted the binding interactions of compounds NCBP-1~NCBP-5 with the protein, encompassing both overall and localized overviews as well as the hydrophobic interactions with the protein (Figure **9A**-**E** and Figure **9a**-**e**). The results demonstrate successful binding of all compounds within the active pocket. All five peptides (NCBP1-NCBP5) exhibited a binding energy of less than −8 kcal/mol with the 4RHB protein. Notably, NCBP-1 exhibits the most favorable binding energy (-12.11 kcal·mol^-1^, Figure **9A** and Table **4**), indicating the strongest affinity with 4RHB, aligning with the *in vitro* antimicrobial screening outcomes [74]. Consequently, we hypothesize that the designed analogues may exert antimicrobial activity *via* binding with the 4RHB protein. This sheds light on the potential mechanisms by which these compounds may act against *E.coli*, and further investigation of the mechanism of activity is needed.

## CONCLUSION

In conclusion, NCBP peptides were synthesized *via* the tag-assisted peptide synthesis (TAPS) strategy, incorporating C-terminal amidation and site-specific mutations. Five derivatives were evaluated against 10 bacterial strains (including Gram-positive and Gram-negative species). NCBP-1, NCBP-2, and NCBP-4 exhibited MIC values of 8-64 μg/mL against all tested strains, with lower values against Gram-negative bacteria (8-32 μg/mL) than Gram-positive counterparts (16-64 μg/mL). This may be attributed to positively charged lysine (Lys) residues introduced during modification, which likely mediate strong electrostatic interactions with negatively charged phosphate groups in the lipopolysaccharide (LPS) layer of Gram-negative bacteria. Such interactions facilitate rapid outer membrane anchoring and penetration, ultimately disrupting the inner membrane and inducing bacterial death. Cytotoxicity assays showed that all derivatives (except NCBP-4) retained >90% cell viability even at 256 μg/mL. Among them, NCBP-1 and NCBP-2 stood out with potent antimicrobial activity (MIC: 8-32 μg/mL against Gram-negative bacteria) and high cell viability (>90% at 256 μg/mL). NCBP-1 also shows moderate anti-inflammatory activity in LPS-stimulated RAW264.7 macrophages. NCBP-1’s antibacterial activity was further validated *via* molecular docking with the outer membrane LPS (4RHB) of *Escherichia coli*. These findings highlight NCBP-1’s excellent antibacterial potential for developing novel agents, particularly against Gram-negative bacterial infections. These promising results warrant further optimization to explore its utility as an alternative or adjunctive antimicrobial.

## STUDY LIMITATIONS

A limitation is that, despite NCBP-1’s promising *in vitro* activity and *in silico* binding, its therapeutic potential is preliminary and requires further *in vivo* validation, mechanistic studies, and related assessments. These promising results warrant further optimization to explore its utility as an alternative or adjunctive antimicrobial.

## Figures and Tables

**Table 1:** Prediction the range of antibacterial activity of the antimicrobial peptides NCBP-1 to NCBP-5.

**Antimicrobial Peptide**	**Sequence**	***CAMPR4***	***DBAASP***				***Hydramp***	
			***E.coli*** ***ATCC 25922***	***K.pneumoniae***	***S.aureus*** ***ATCC 25923***	***Hemolysis***	***Pr*** ***(AMP)***	***Pr(MIC)*** ***E.coli***
NCBP-1	KPWLRVALCPG	0.88	N	N	N	N	0	0
NCBP-2	KKWLRVALCKG	1	Active	Active	N	N	0.14	1
NCBP-3	KKWLRVALKKG	1	Active	Active	N	N	1	1
NCBP-4	KKWLRVWLCKG	0.99	Active	Active	N	N	1	0.99
NCBP-5	KKWLRVWLKKG	0.97	Active	Active	Active	N	1	1

**Note: Remark:** Multi-Database Prediction of Antimicrobial Peptide Potential. **DBAASP:** N: No activity; Active: active peptides are defined as those with a MIC value < 25 μg/mL. **Hemolysis:** peptides with a hemolytic activity > 40% at a concentration < 40 μg/ml are defined as hemolytic peptides.

**Table 2:** Prediction and physico-chemical properties of the antimicrobial peptides NCBP-1 to NCBP-5.

**Antimicrobial peptide**	**Sequence**	**HeliQuest**				**Similarity Search**		
		**Length**	**Charge**	**Hydrophobicity**	**Hydrophobic moment**	**APD3**	**CAMPR3**	**DRAMP**
NCBP-1	KPWLRVALCPG	11	2	0.742	0.289	No	No	No
NCBP-2	KKWLRVALCKG	11	3	0.431	0.241	No	No	No
NCBP-3	KKWLRVALKKG	11	5	0.201	0.467	No	No	No
NCBP-4	KKWLRVWLCKG	11	4	0.607	0.417	No	No	No
NCBP-5	KKWLRVWLKKG	11	5	0.377	0.640	No	No	No

**Note: Remark:** Physico-chemical properties of peptides by Heliquest software: (http://heliquest.ipmc.cnrs.fr/).

**Table 3:** Minimum inhibitory concentrations (MIC) of Antimicrobial peptides NCBP-1 ~ NCBP-5 against five gram-negative bacteria and five gram-positive bacteria.

**NCBP** **(1~5)**	**Minimal Inhibitory Concentration (MIC) (μg·mL^-1^)**												
	**Gram-negative**					**Gram-positive**					**Average**		
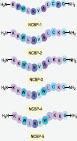	*E.Coli* *ATCC 25922*	*K.a pneumoniae* *ATCC 13883*	*S.flexneri* *ATCC 12022*	*S. enteritidis* *CMCC(B) 50335*	*S.typhimurium* *ATCC14028*	*S.aureus* *ATCC 25923*	*S.aureus* *ATCC 6538*	*S.aureus* *CMCC(B) 26003*	*MRSA* *ATCC 4330*	*E.faecalis* *ATCC 29212*	*GMG-*	*GMG+*	*GMall*
Polymyxin B	4	8	1	32	4								
Vancomycin						0.5	0.5	0.5	0.5	2			
NCBP-1	8	16	8	16	16	32	64	16	64	32	13.93	36.76	21.11
NCBP-2	32	32	16	32	16	32	64	16	64	64	24.25	32	42.22
NCBP-3	128	128	64	128	128	128	128	128	128	128	111.43	128	119.43
NCBP-4	32	16	16	32	32	32	32	16	32	32	24.25	27.86	25.99
NCBP-5	32	128	16	32	32	32	128	16	64	64	36.76	48.5	42.22

**Table 4:** The docking binding energy and MIC against *E.coli* for Antimicrobial peptides NCBP-1 ~ NCBP-5.

**Peptide**	**Binding Energy** **(kcal·mol^-1^)**	**Hydrogen Bonds**	**Conjoint Residues**	**MIC** **(μg·mL^-1^)**
NCBP-1	-12.11	14	GLN-140,ASP-141,TYR-314,ARG-326,ASN-328,ASP-330,THR-356,SER-360,SER-373,ASP-395,	8
NCBP-2	-8.678	11	ASP-85,TYR-244,TYR-314,TYR-347,SER-349,THR-351,ASP-352,ASN-755,MET-772,SER-775	32
NCBP-3	-8.573	12	TYR-244(2),ASN-345,SER-349,GLY-731,GLU-733,ASN-755,MET-772,SER-775,ILE-777,	128
NCBP-4	-9.519	11	LYS-84,GLN-140,LYS-234,TYR-244(2),TYR-314,ASP-330,ASN-345,THR-356,MET-772, SER-775,	32
NCBP-5	-8.304	13	LYS-84,ASN-316,ASP-330,LYS-346,GLY-348,GLU-733,GLU-757,GLU-771	32

## Data Availability

All data generated or analyzed during this study are included in this published article.

## LIST OF ABBREVIATIONS

SPPS: Solid Phase Peptide Synthesis
MDR: Multidrug-Resistant
MRSA: Methicillin-Resistant *Staphylococcus aureus* (MRSA)
AMP: Antimicrobial Peptides
LPS: Lipopolysaccharide
RP-HPLC: Reversed-Phase High-Performance Liquid Chromatography
MIC: Minimal Inhibitory Concentration
CCK-8: Cell Counting Kit-8
DCM: Dichloromethane
DMP: N,N-Dimethylpropyleneurea
HOBT: 1-Hydroxybenzotriazole
TFA: Trifluoroacetic Acid
EDT: Ethanedithiol
TIS: Triisopropylsilane
DMSO: Dimethyl sulfoxide
MHA: Mueller-Hinton Agar
MHB: Mueller-Hinton Broth
MCF: McFarland Standard
OD: Optical Density
